# Gender Differences in Fear and Risk Perception During the COVID-19 Pandemic

**DOI:** 10.3389/fpsyg.2021.689467

**Published:** 2021-08-05

**Authors:** Abdelaziz Alsharawy, Ross Spoon, Alec Smith, Sheryl Ball

**Affiliations:** ^1^Department of Economics, Virginia Tech, Blacksburg, VA, United States; ^2^School of Neuroscience, Virginia Tech, Blacksburg, VA, United States

**Keywords:** gender differences, COVID-19, fear, health behavior, beliefs, risk perception, affect heuristic

## Abstract

The COVID-19 pandemic has led many people to suffer from emotional distress. Previous studies suggest that women process and express affective experiences, such as fear, with a greater intensity compared to men. We administered an online survey to a sample of participants in the United States that measures fear of COVID-19, perceptions about health and financial risks, and preventative measures taken. Despite the empirical fact that men are more likely to experience adverse health consequences from COVID-19, women report greater fear and more negative expectations about health-related consequences of COVID-19 than men. However, women are more optimistic than men regarding the financial consequences of the pandemic. Women also report more negative emotional experiences generally during the pandemic, particularly in situations where other people or the government take actions that make matters worse. Though women report taking more preventative measures than men in response to the pandemic, gender differences in behavior are reduced after controlling for fear. These results shed light on how differences in emotional experiences of the pandemic may inform policy interventions.

## Introduction

The consequences of COVID-19 transcend public health. The pandemic has profoundly affected economic activity, social interactions, and emotional wellbeing. Despite the universality of the pandemic, experience with previous natural disasters suggests that its impact may vary across individuals. Gender, age, socioeconomic status, and affective responses all influence how people are affected by catastrophic events (Neumayer and Plümper, 2007; Taylor et al., 2008; Eckel et al., 2009; Ibuka et al., 2010; Huang et al., 2013; Callen et al., 2014; Jang et al., 2020). For example, among earthquake victims in Turkey, women were more likely to recall panicking during the crisis (Yilmaz et al., 2005). Moreover, women were also more likely to report fear of disasters, such as landslide or flooding in Taiwan (Ho et al., 2008), and to worry about serious negative consequences of climate change in Sweden (Sundblad et al., 2007).

Gender differences are common in self-reported emotional experiences. Women report greater affective intensity (Fujita et al., 1991) and experience negative emotions, such as fear more frequently (Brebner, 2003; Fischer et al., 2004). The COVID-9 pandemic is no exception. In recent surveys conducted in the United States, Cuba, and China, women reported greater fear and stress associated with the pandemic (Broche-Pérez et al., 2020; Fitzpatrick et al., 2020; Liu et al., 2020; Park et al., 2020). Early research on the impact of the COVID-19 pandemic suggests that local COVID-19 infection rates (Bu et al., 2020) and fear of the virus decrease risk taking (Alsharawy et al., 2020) and predict adherence to prevention measures (Harper et al., 2020; Müller and Rau, 2021). In addition, across eight different countries, women had a greater perception of the severity of the COVID-19 pandemic and greater adherence to prevention measures (Galasso et al., 2020).

Interestingly, these differences run counter to sex differences in the health consequences of the pandemic. Though disease prevalence is roughly equal between males and females, males are more likely to experience serious health consequences and to die from COVID-19 (Bhopal and Bhopal, 2020; Gebhard et al., 2020; Jin et al., 2020; Peckham et al., 2020). A recent meta-analysis indicates that, conditional on a positive diagnosis, males have roughly a 40% greater mortality risk from COVID-19 and are nearly three times more likely to be admitted to hospital intensive treatment units (Peckham et al., 2020).

We surveyed nearly 1,500 people across the United States to measure emotions, behaviors, and expectations associated with the COVID-19 pandemic. We hypothesized that women would report higher levels of fear, and this would motivate higher adherence to COVID-19 prevention measures, such as washing hands or physical distancing. Similarly, we explore whether pro-sociality increases adherence to mitigation strategies. Finally, based on the previous studies of natural disasters, we also expected that women would report greater concern about the negative consequences of the crisis.

## Materials and Methods

In April 2020, we administered a repeated cross-sectional survey to a random sample of around 1,500 people residing in the United States on Amazon Mechanical Turk (MTurk). We collected a third of our data every two weeks starting on April 2, 2020. There were approximately 200,000 confirmed COVID-19 cases in the United States at the time of our first sample; this number was tripled in the following two weeks and reached over 1 million cases by our third wave. The number of United States deaths from COVID-19 was less than 4,000 at the time of our initial sample, reached about 26,000 two weeks later, and passed 50,000 around the time of our third sample wave (Coronavirus disease 2019 (COVID-19) Situation Report – 73, 2020; Coronavirus disease 2019 (COVID-19) Situation Report – 87, 2020; Coronavirus disease 2019 (COVID-19) Situation Report – 101, 2020). To determine local COVID-19 infection rates, we matched participants’ ZIP codes to counties (using a publicly available ZIP code database)^1^ and obtained county-level data on population and COVID-19-related deaths from the COVID-19 Data Repository by the Center for Systems Science and Engineering (CSSE) at Johns Hopkins University^2^ (Dong et al., 2020).

Our survey captured self-reported fear of COVID-19 and adherence to preventative health behavior. Participants also indicated their perceptions of health and financial risks in the form of probabilistic beliefs about the percent chance that (1) they or a household member will lose a job due to the pandemic, (2) total household income will decrease over the next 12 months, (3) they or someone close will develop COVID-19, and (4) they or someone close will die from COVID-19. To elicit these beliefs, we adapted question formats that were validated against realizations of the same events (Manski, 2004). We also elicited anticipated negative emotions after people or institutions make decisions that make matters worse during a crisis. The full list of survey questions is provided in the Supplementary Material (see Sections 1.4 and 1.5). The survey included other measures that are discussed in a companion paper on fear of COVID-19 and economic preferences, which finds that risk and time preferences varied significantly with fear of COVID-19 and the association weakening over time (Alsharawy et al., 2020). We designed this survey in the early weeks of the pandemic to capture individual and socioeconomic characteristics (22 questions), economic preferences from the Global Preference Survey (10 questions; Falk et al., 2016, 2018), unincentivized risky lottery preference (Eckel and Grossman, 2002), and trust (nine questions adapted from Global Preference Survey, Socio-Economic Panel Study and World Value Survey, Inglehart, 2004; Wagner et al., 2007; Falk et al., 2016, 2018). In addition, we surveyed participants on their behavior and beliefs with regard to the pandemic (14 questions), and expectations about the emotions they would experience if people/institutions made wrong decisions in response to a crisis (4 questions). In this study, we explore gender differences in behavior, beliefs, and expectations with regard to the pandemic.

We set an initial criterion in our first wave of master status for MTurk workers. For subsequent waves, we then dropped this requirement, due to difficulties in collecting our predetermined sample size of 500 per wave, while still requiring a 99% or higher approval rating and at least 5,000 approved Human Intelligence Tasks. Due to random sampling from eligible participants, our sample is not strongly balanced across genders (690 women and 794 men). Moreover, 71% of our sample participants took the survey only once, so there is not a sufficient number of repeaters in our sample to investigate individual changes over time. We therefore combine the three waves, and in our regression analyses, we include controls for wave-specific effects. There are some differences in survey responses across genders on factors, such as age, political orientation, and education (see Supplementary Table S2). Similar to other studies analyzing survey responses (Dohmen et al., 2011; Falk et al., 2018), we control for these differences statistically using individual-level characteristics to establish the robustness of our findings: age, age-squared, indicator for race (Caucasian) or origin (Hispanic), self-reported high household income relative to others in one’s community, working full time, education level, parents receiving a bachelor’s degree, smoking behavior, and frequency of attending religious services. In addition, we control for occupation adapting a categorization from the Census classification as outlined in the Supplementary Material (2010 Census Occupational Classification, 2016). Our regression analysis also controls for the state in which the participant resided, in which of the three survey waves they participated, and the local (county) death rate of COVID-19 (per 100,000 population) (Bu et al., 2020).

## Hypotheses

Building on previous findings of women reporting higher frequency of negative emotions (Brebner, 2003; Fischer et al., 2004), we hypothesized that women would report higher fear levels of COVID-19 in the early weeks of the pandemic (question 60 in our survey; see Supplementary Material). Confirming this hypothesis would bolster the credibility of recent findings that are reported in surveys in the United States and Cuba (Broche-Pérez et al., 2020; Fitzpatrick et al., 2020).

*H1*: Women, compared to men, report higher fear of the COVID-19 pandemic.

Since emotional experiences are widely believed to affect behavior (Forgas, 1995; Loewenstein et al., 2001; Barrett, 2006; Baumeister et al., 2007; Van Kleef, 2009) and the pandemic evoked emotional responses in many ways (Alsharawy et al., 2020; Taylor et al., 2020a,b), we were interested in whether gender differences in adherence to the disease’s prevention measures were mediated by fear of COVID-19. In particular, we hypothesized that controlling for self-reported fear of the pandemic would weaken the relationship between gender and adherence to preventative measures (measured in question 54 in our survey; see Supplementary Material).

*H2*: Controlling for fear of COVID-19 weakens observed gender differences in adherence to prevention measures.

Worries about the health-related dangers of the COVID-19 have been strongly linked to distress (Taylor et al., 2020a), so we explored gender differences in expectations about COVID-19-related outcomes. In particular, we elicited participants’ beliefs of experiencing both health and financial hardships as a result of the pandemic. Since women tend to report greater affective intensity (Fujita et al., 1991) and consistent with the affect heuristic (Finucane et al., 2000; Loewenstein et al., 2001; Slovic and Peters, 2006; Slovic et al., 2007), we hypothesized that women have more negative perceptions about the COVID-19 risks (measured in questions 56–59 in our survey; see Supplementary Material). Moreover, we explore whether gender differences extend to expectations about experiencing negative emotions when decisions made by other people, the government, the media, or autonomous devices make matters worse during a crisis (measured in questions 43–46). We hypothesized that women expect to experience stronger negative emotions in such cases.

*H3A*: Women, compared to men, report higher expectations of negative health- and financial-related consequences of the COVID-19 pandemic.

*H3B*: Women, compared to men, report higher expectations of experiencing negative emotions in a crisis when decisions made by other people, institutions, or autonomous devices make matters worse.

## Results

**Figure 1:**
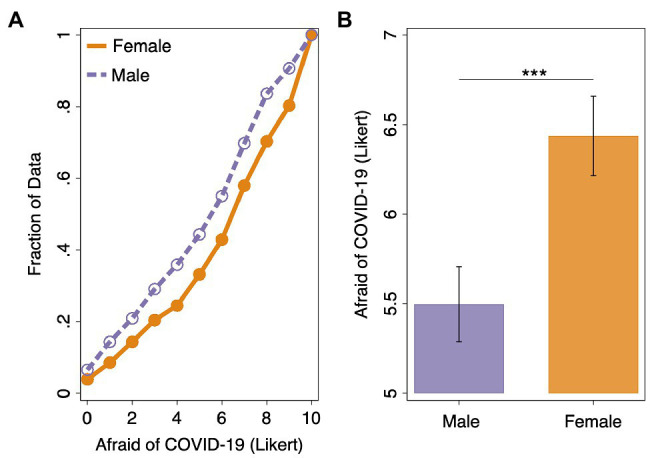
**(A)** Empirical cumulative distribution function for fear of COVID-19 by gender (11-point Likert question with response alternatives ranging from “Not at all afraid” at 0 to “Very afraid” at 11). **(B)** Average fear of COVID-19 by gender (error bars represent 95% confidence intervals). Wilcoxon rank-sum tests: ^***^p < 0.001.

First, we investigate whether emotional responses to the pandemic, in particular fear, differed across self-reported gender. Confirming our first hypothesis, women reported higher fear of the COVID-19 pandemic compared to men in our pooled sample ($\mu_{difference}=\;\;0.939,$Wilcoxon rank-sum test: *p* < 0.001; see Figure 1). In addition to reporting the results of the widely used nonparametric Wilcoxon rank-sum test that probe for differences in central tendency, we report in Supplementary Table S1 the results of two additional statistical analyses: two-sided *t*-tests (parametric: central tendency) and Epps-Singleton tests (nonparametric: distributional characteristics). Importantly, this gender difference in fear of the pandemic is robust across statistical tests. When we examine the distribution of the Likert scale responses, we find that women were more than twice as likely to report extreme levels of fear than men. Nearly 20.0% of women chose the highest available value for fear of the pandemic, compared to around 9.3% of men. This finding of increased fear of the pandemic among women is also robust in multiple regression analysis controlling for state and survey-wave fixed effects (β = 0.963, *p* = 0.001) and to individual-specific controls, including age, ethnicity, occupation, employment status, political orientation, smoking behavior, self and parent’s education, self-reported income, and a self-reported measure of cognitive ability (β = 0.654, *p* = 0.014; see Supplementary Table S3). As reported in our companion paper, we use the local death rate as a proxy for the intensity of individual experience of the pandemic. The local death rate was positively and significantly associated with fear of COVID-19 (Alsharawy et al., 2020). These results also hold when we standardize (z-score) the Likert response for each individual to account for differences in response styles (Fischer and Milfont, 2010; results available upon request). Moreover, when we include the interaction between the gender and each of the two waves, we find that the rate by which self-reported fear declined over time was similar across genders (*p* > 0.100; result available upon request).

Second, we turn to self-reports of whether respondents adopted recommended preventative health behaviors in response to the COVID-19 pandemic. We examine the following: (1) hand washing, (2) using hand sanitizer, (3) avoiding touching one’s face, (4) cleaning and disinfecting surfaces in the home, (5) wearing a face mask, and (6) practicing physical distancing. Using an Ordered Logit regression where the dependent variable is the number of preventative measures taken (see Table 1), we find that women adopted significantly more preventative measures than men (OR = 1.355, *p* = 0.003). This result is robust to the inclusion of individual-level controls (OR = 1.314, *p* = 0.010). Holding all other variables constant, this model suggests that the odds of following all six preventative measures are 1.314 greater for women than men. Interestingly, when we include self-reported fear of the COVID-19 pandemic as a predictor, the gender difference result no longer holds (OR = 1.104, *p* > 0.100). Instead, the coefficient for fear of the COVID-19 pandemic is positive and statistically significant (OR = 1.255, *p* < 0.001). With a one (Likert)-unit increase in self-reported fear of the pandemic while holding other variables constant, the odds of adhering to all six health behaviors versus the combined other categories are greater by a factor of 1.255. Again, these results are robust to the inclusion of individual-level controls (see Table 1). Our findings suggest that gender differences in behavioral responses, both in our and in other studies (e.g., Galasso et al., 2020), are driven by emotional responses to the COVID-19 pandemic. There is recent evidence suggesting that social preferences, in particular pro-sociality, increases adherence to prevention measures (Campos-Mercade et al., 2021). Moreover, in our companion paper, we report that fear of COVID-19 and altruism are positively and significantly associated (Alsharawy et al., 2020). In our survey, we capture an experimentally validated measure of altruism (question 26 in our survey; see Supplementary Material; Falk et al., 2016, 2018), and we also find that pro-sociality is positively and significantly associated with compliance to preventative measures (OR = 1.173, *p* < 0.001; see Table 1; Campos-Mercade et al., 2021). Importantly, however, the positive statistical significance between fear of COVID-19 and compliance to preventative measures remains robust despite controlling for altruism (OR = 1.236, *p* < 0.001). In addition, we find a similar result for local COVID-19 infection rates. In particular, the positive relationship between local death rate and the number of prevention measure taken (OR = 1.009, *p* = 0.004) is weakened when we control for fear of COVID-19 (OR = 1.006, *p* = 0.044). These results confirm the importance of affective responses, namely fear, in behavioral responses during a crisis, like the COVID-19 pandemic.

**Table 1:** Number of preventative measures taken in response to COVID-19 (Ordered Logit Regression).

Dependent variable	(a)Preventative measures taken	(b)Preventative measures taken	(c)Preventative measures taken	(d)Preventative measures taken	(5)Preventative measures taken
Female	1.3546^***^	1.1043	1.3141^***^	1.1419	0.992
	(0.1397)	(0.1249)	(0.1392)	(0.1451)	(0.1433)
Afraid of COVID-19	–	1.2549^***^	–	1.245^***^	1.2357^***^
		(0.0262)		(0.0262)	(0.0269)
Wave 2	2.0753^***^	2.4407^***^	2.0755^***^	2.4736^***^	2.5451^***^
	(0.1826)	(0.2338)	(0.1789)	(0.2531)	(0.2716)
Wave 3	3.193^***^	4.1211^***^	2.9551^***^	3.761^***^	3.8504^***^
	(0.4561)	(0.5525)	(0.4651)	(0.5781)	(0.6041)
Altruism	–	–	–	–	1.1731^***^
					(0.0278)
Local death rate	–	–	1.0089^***^	1.0062^**^	1.0063^**^
			(0.0031)	(0.003)	(0.0032)
Cognitive ability	–	–	0.9779	0.9975	0.9946
			(0.0239)	(0.0243)	(0.024)
Liberal	–	–	1.1104^***^	1.0572^***^	1.0475^***^
			(0.0228)	(0.0197)	(0.0188)
Additional controls	No	No	Yes	Yes	Yes
State fixed effects	Yes	Yes	Yes	Yes	Yes
Observations	1,484	1,484	1,484	1,484	1,484

*The six measures are as follows: (1) washing hands more frequently, (2) using hand sanitizers more frequently, (3) make more effort to avoid touching face, (4) cleaning and disinfecting surfaces in home more than usual, (5) wearing a face mask, and (6) engaging in physical distancing. Odds ratios reported. Standard errors (clustered at the state level) in parentheses. Additional controls included age, age-squared, and indicators for race (Caucasian) and origin (Hispanic), occupation (eight categories), self-reported same or high household income relative to others in one’s community, working full time, education level, parents receiving a bachelor’s degree, smoking behavior, and frequency of attending religious services*.

*** *p < 0.01;*

** *p < 0.05*.

*Table was created using asdoc, a Stata program written by Shah (2020)*.

We run alternative specifications investigating each of the six prevention measures separately, using a series of Logit regressions that control for state and survey-wave fixed effects and individual-level characteristics (see Supplementary Tables S4 and S5). We find that women, compared to men, were significantly more likely to report making an effort to avoid touching one’s face (*OR* = 1.483, *p* = 0.030), to clean and disinfect surfaces (*OR* = 1.553, *p* = 0.003) and to engage in physical distancing (*OR* = 1.661, *p* = 0.036). These associations become weaker when we control for fear of COVID-19. Though women are still significantly more likely to report cleaning and disinfecting surfaces (*OR* = 1.409, *p* = 0.025) after controlling for fear, gender differences in making an effort to avoid touching one’s face or engaging in physical distancing shrunk when including fear as a covariate (*OR* = 1.311, *p* = 0.172; *OR* = 1.431, *p* = 0.216, respectively). Importantly, however, we find that fear of COVID-19 is strongly associated with adherence to each of our six preventative measures (*OR* > 1.189, *p* < 0.001 for all tests). This result holds even after controlling for altruism, which was positively and significantly associated with compliance to all preventative measures except washing hands more frequently (*OR* > 1.079, *p* < 0.010; results available upon request). Again, these findings provide evidence in favor of our second hypothesis and demonstrate the importance of fear of COVID-19 in predicting preventative behavior (Harper et al., 2020).

**Figure 2:**
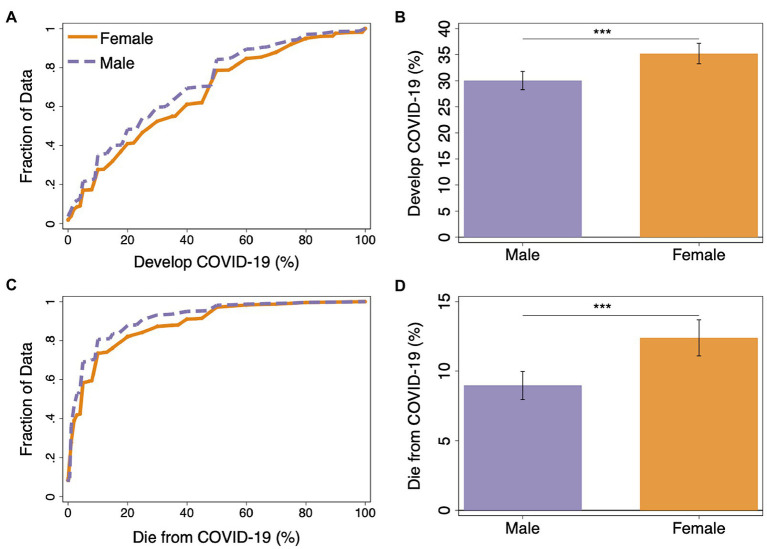
**(A)** Empirical cumulative distribution function (eCDF) for self-reported beliefs about the likelihood of oneself or someone close developing COVID-19 (develop COVID-19). **(B)** Average self-reported beliefs of developing COVID-19. **(C)** eCDF for the self-reported beliefs about the likelihood of oneself or someone close dying from COVID-19 (Die from COVID-19). **(D)** Average self-reported beliefs of dying from COVID-19. Data are split by gender (error bars represent 95% confidence interval). Wilcoxon rank-sum tests: ^***^p < 0.001.

Next, we explore whether there were gender differences in self-reported probabilistic beliefs about the likelihood of experiencing health and financial hardships due to the COVID-19 pandemic. We find that beliefs about the likelihood of health consequences of COVID-19 differed between men and women. Contrary to the empirical observation that men are more likely to experience severe illness or die as a result of COVID-19 (Bhopal and Bhopal, 2020; Gebhard et al., 2020; Jin et al., 2020; Peckham et al., 2020), men reported systematically lower expectations of negative health-related consequences of the pandemic. Women, on average, reported a 5.2% higher chance that they or someone close would develop COVID-19 compared to men and 3.4% higher chance of oneself or someone close dying from COVID-19 (see Figure 2). The distribution of beliefs about the likelihood of experiencing health hardships indeed differed significantly for both contracting COVID-19 and dying from COVID-19 (Wilcoxon rank-sum test: *p* < 0.001 and *p* < 0.001, respectively). Men were more likely to indicate a low likelihood of contracting COVID-19, with 35.0% of men indicating a 10% or less chance, compared to 27.7% of women. This difference holds when we look at beliefs about the likelihood of dying from COVID-19, with 73.5% of women indicating a 10% or less chance of that scenario relative to 80.6% for men. Taken together, this means that we find that women report higher fear of the COVID-19 pandemic and stronger negative beliefs about health consequences. The finding that women believe there are significantly higher chances of developing or dying from COVID-19 is robust to the inclusion of state and survey-wave fixed effects and individual-level controls (β = 3.341, *p* = 0.009; β = 2.425, *p* = 0.022, respectively; see Supplementary Table S6).

Despite the absence of central tendency gender differences in the expectation of experiencing financial hardships, such as job loss or decline in income ($\mu_{difference}=\;\;0.793$ and $\mu_{difference}=\;\;-1.912;$ Wilcoxon rank-sum test: *p* = 0.354 and *p* = 0.137, respectively; see Supplementary Figure S1 and Supplementary Tables S1 and S6), tests that probe more broadly to distributional characteristics (Epps and Singleton, 1986; Goerg and Kaiser, 2009) reveal some variations in the spread of expectations in the probabilistic beliefs about the likelihood of job loss and income loss across genders (see Supplementary Table S1). These differences can be attributed to lower expectations of experiencing financial hardship among women than among men. For example, 48.0% of women indicated a 10% or less chance of job loss compared to only 42.8% of the men. Furthermore, 32.3% of women indicated a 10% or less chance of experiencing income loss compared to only 27.1% of men. Thus, we find significant gender differences in expectations regarding health, but not financial consequences of the COVID-19 pandemic, partially confirming Hypothesis 3A. Moreover, both women and men predicted a lower chance of job loss due to the COVID-19 pandemic than of income loss ($\mu_{difference}=\;\;-13.465;$ $\mu_{difference}=\;\;-16.171;$ Wilcoxon signed-rank test: *p* < 0.001). Overall, survey responders anticipated a 26.6% chance of job loss and a 41.5% chance of a decline in household income.

**Figure 3:**
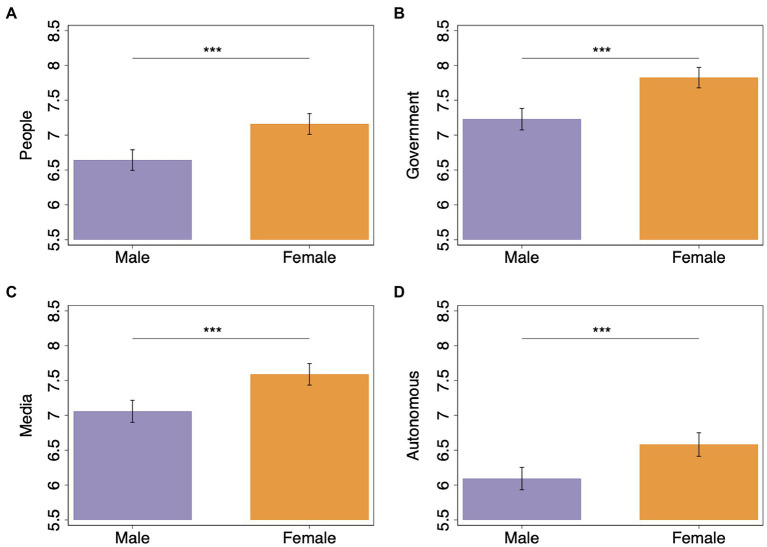
Expected negative emotional experience (e.g., sadness or anger) in a hypothetical scenario where **(A)** other people **(B)** the government **(C)** the media, or **(D)** an autonomous system take actions that make matters worse in a crisis (11-point Likert question with response alternatives ranging from “Not at all” at 0 to “A great deal” at 11). Data are split by gender (error bars represent 95% confidence interval). Wilcoxon rank-sum test: ^***^p < 0.001.

We also elicited the extent to which survey responders experience negative emotions, such as sadness or anger, when decisions made by other people, the government, the media, or autonomous devices might make matters worse during a crisis. Across all these measures, we find that women anticipated experiencing significantly more intense negative emotions than men ($\mu_{difference_{people}}=\;\;0.517,$ $\mu_{difference_{government}}=\;\;0.594,$ $\mu_{difference_{media}}=\;\;0.528,$ and $\mu_{difference_{autonomous}}=\;\;0.488,$ Wilcoxon rank-sum test: *p* < 0.001 for all four measures; see Figure 3). We find that women reported not only higher fear of the COVID-19 pandemic but also a higher tendency to experience negative emotions during crises in general, in particular as a result of unfavorable actions taken by people, institutions, and devices. This confirms Hypothesis 3B. After including state and survey-wave fixed effects and individual-level controls in multiple regression analysis, the intensity of negative emotions that women report experiencing during crises was significantly greater than that of men (people: β = 0.356, *p* = 0.007; government: β = 0.463, *p* = 0.002; media: β = 0.385, *p* = 0.016; autonomous: β = 0.315, *p* = 0.016; see Supplementary Table S7).

## Discussion

We investigated gender differences in the intensity of experiencing negative emotions, namely fear, in response to the COVID-19 outbreak. In our study, women report higher fear of the COVID-19 pandemic compared to men. Gender differences in preventative health behaviors disappeared once we controlled for emotional experiences, suggesting that fear of the COVID-19 pandemic, and not gender per se, drives behavioral differences. Women report more negative perceptions about the pandemic’s health, but not economic, risks. Thus, our findings on health risks are consistent with the affect heuristic: the notion that emotional experience shapes the perception of risk (Finucane et al., 2000; Loewenstein et al., 2001; Slovic and Peters, 2006; Slovic et al., 2007; Skagerlund et al., 2020). Maladaptation in face of threats has been linked to overconfidence and positive illusion (Johnson and Levin, 2009). Our results may thus be related to domain specific overconfidence/underconfidence (Klayman et al., 1999; Johnson and Fowler, 2011), with men being more overconfident and women being more underconfident (Barber and Odean, 2001; Bengtsson et al., 2005; Johnson et al., 2006). Gender stereotypes are manifested in women’s emphasis on care compared to men’s emphasis on agency (Ellemers, 2018), while social concerns have been argued to modulate overconfidence (Burks et al., 2013). Our results may suggest that gender stereotyping may play a role in the existence of a gap between negative perception of health but not financial risks. In addition, structural labor market concerns, such as the gender wage gap, as well as workplace- and occupation-specific factors (Blau and Kahn, 2017; Wiswall and Zafar, 2018), may also contribute to the observed differences in perceptions of health and financial risks. While we account for occupation in our analyses, the broad classifications utilized (see Materials and Methods section) are somewhat limited. For example, our observation that women have less extreme views of the financial consequences of the pandemic could result from their self-selection into jobs with greater work flexibility and job stability (Wiswall and Zafar, 2018). Nonetheless, we find that women report stronger negative emotions resulting from crises in general, as a result of unfavorable actions taken by, for example, other people and the government. Our results contribute to the literature on gender differences in economic preferences, which finds that women are typically more risk averse (Eckel and Grossman, 2002; Dohmen et al., 2011; Charness and Gneezy, 2012) and less likely to prefer competition (Niederle and Vesterlund, 2007; Buser et al., 2014). As in our study, these gender differences may reflect state dependent variation, rather than stable traits (Frey et al., 2017; Pedroni et al., 2017; Mata et al., 2018).

One limitation of our study is the reliance on questionnaire responses. This seemed a reasonable compromise between our desire to obtain data at the beginning of the COVID-19 event in the United States and the need to keep both participants and experimenters safe. In fact, recent empirical work on preference elicitation suggests that self-reported preferences are generalizable and may be more stable across time compared to incentivized behavioral measures (Frey et al., 2017; Pedroni et al., 2017; Mata et al., 2018). Our questionnaire was designed in the early days of the pandemic and prior to the development of the multiple-scale measures of fear of COVID-19 (Ahorsu et al., 2020; Feng et al., 2020; Mejia et al., 2020). Nonetheless, our survey question that captures fear of the pandemic matches one of the items with a strong factor loading in the commonly used fear of COVID-19 scale (Ahorsu et al., 2020). The finding of gender differences in fear of the pandemic is not unique to the early days of the pandemic (Alsharawy et al., 2021). In addition, though our study relies on correlations between survey measures, and therefore, our results cannot be interpreted as causal, we demonstrate that our findings are robust.

Our study suggests avenues for future study for researchers interested in effective crisis management. To mitigate the severity of a crisis, for example, policy makers sometimes employ fear messaging, or scare tactics, to promote adherence to prevention measures. Our results suggest that this approach may have differential impact depending on gender, since women report higher fear. Furthermore, scare tactics may also have unintended consequences, such as increasing message avoidance (Kok et al., 2014) or exacerbating existing stressors (Stolow et al., 2020). Messaging strategies that emphasize the pro-social implications of preventative measures, that focus on evidence-based health communications, or that “nudge” behavior in a contextually appropriate manner (Kreuter and Wray, 2003; Campos-Mercade et al., 2021; Heffner et al., 2021; Milkman et al., 2021) without increasing psychological distress may be preferred during health crises.

## Data Availability Statement

Data and analysis script that support the findings of this study is available through the Open Science Framework at https://osf.io/drhfw.

## Ethics Statement

The studies involving human participants were reviewed and approved by The Institutional Review Board of Virginia Tech. The participants provided their written informed consent to participate in this study.

## Author Contributions

All authors contributed to the conception and design of the study, manuscript revision, read, and approved the submitted version. AA and RS organized the database. AA performed the statistical analysis and wrote the first draft of the manuscript. AA, AS, and SB wrote the sections of the manuscript.

## Conflict of Interest

The authors declare that the research was conducted in the absence of any commercial or financial relationships that could be construed as a potential conflict of interest.

## Publisher’s Note

All claims expressed in this article are solely those of the authors and do not necessarily represent those of their affiliated organizations, or those of the publisher, the editors and the reviewers. Any product that may be evaluated in this article, or claim that may be made by its manufacturer, is not guaranteed or endorsed by the publisher.

## Footnotes

^1^ www.unitedstateszipcodes.org/zip-code-database

^2^ https://github.com/CSSEGISandData/COVID-19
