# Supplementary material for: Gender Differences in Fear and Risk Perception During the COVID-19 Pandemic
Source: Front Psychol. 2021 Aug 5;12:689467. doi: 10.3389/fpsyg.2021.689467 (PMC8375576; doi:10.3389/fpsyg.2021.689467)
Supplement: Supplementary file 1 [file Data_Sheet_1.pdf]

Supplementary Material

1.1 Supplementary Text

Occupation classification

We categorized each of the free form self-reported occupations into one of 22 broad occupation categories, based upon the 2010 census occupational classification. To have adequate number of observations within each category, we then assign occupations into broader categories, again adapted from the 2010 census occupational classification, while creating a separate category for health care workers (technical practitioners and support staff). This method assigned participants to one of eight occupation categories: 1) Healthcare, 2) Management, business, and financial operations, 3) Natural resources, construction, and maintenance 4) Production, transportation, and material moving, 5) Professional and related -not healthcare, 6) Sales and office, 7) Service -not healthcare, and 8) Other. Our last category includes responders whose occupations could not be matched to one of the other categories, such as students, homemakers, unemployed/retired, or participants indicating that their occupation is being an MTurk worker.

Statistical tests for gender differences in survey responses

In this section, to further investigate the robustness of our findings, we compare the results of the Wilcoxon rank-sum tests (non-parametric: central tendency) from the main manuscript to two-sided t-tests (parametric: central tendency) and Epps-Singleton tests (non-parametric: distributional characteristics).

1.2 Supplementary Figures

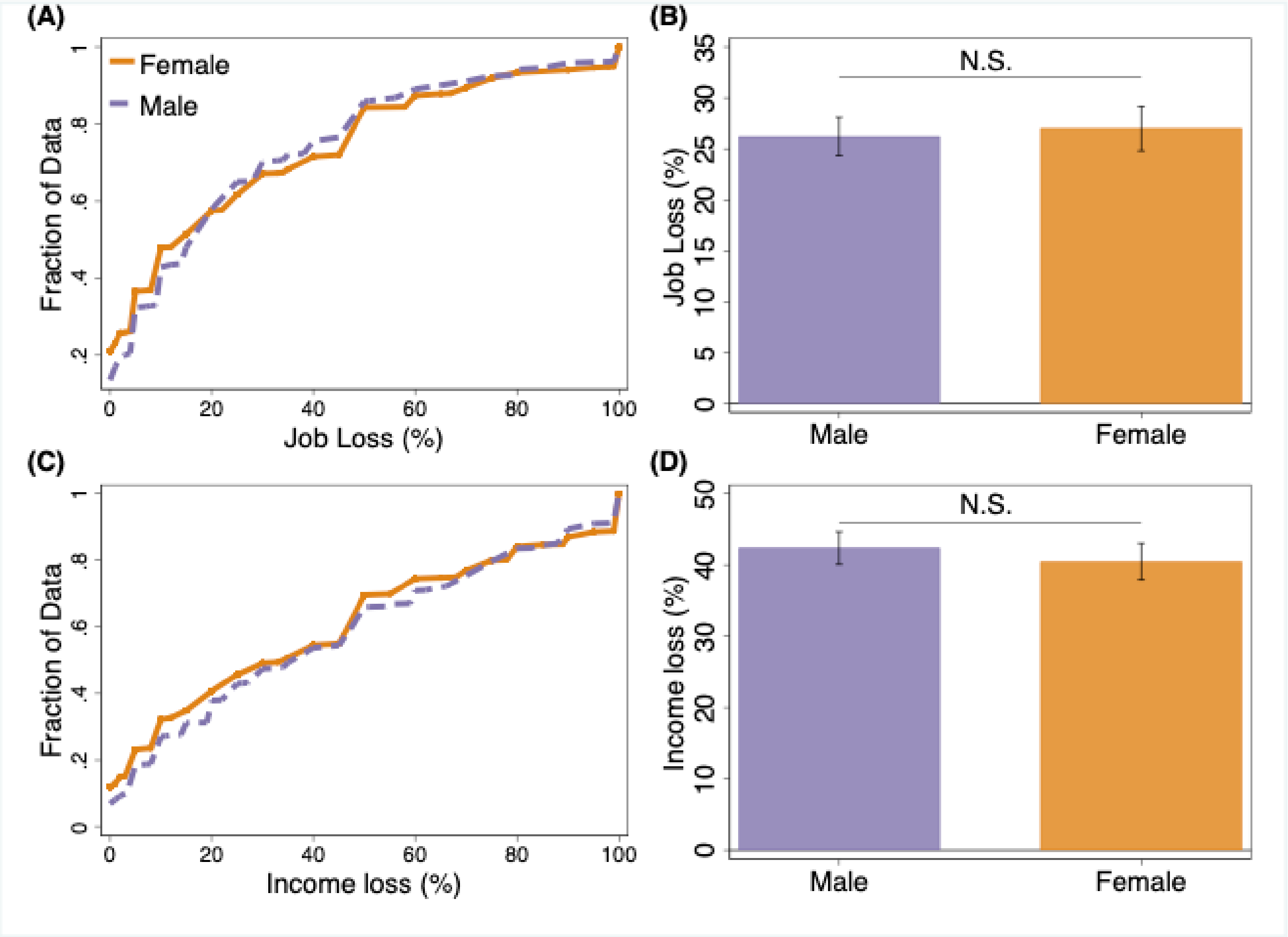

Supplementary Figure S1: (A) Empirical cumulative distribution function (eCDF) for self-reported beliefs about the likelihood of oneself or another member of one's household losing a job or a business in the next 12 months (job loss). (B) Average self-reported beliefs of job loss. (C) eCDF for the self-reported beliefs about the likelihood of one's total household income to decline in the next 12 months (income loss). (D) Average self-reported beliefs of income loss. Data is split by gender (Error bars represent confidence Interval). Wilcoxon rank-sum test: N.S. Non-significant.

1.3 Supplementary Tables

Supplementary Table S1: Statistical tests for gender differences in survey responses

Test for gender differences in		Mean difference (Women−Men):	Wilcoxon Rank-sum test	Two-sided t-test P-value	Epps-Singleton distribution test P-value
Fear of the COVID-19 pandemic		0.939	P<0.001	P<0.001	P<0.001
Chance that they or someone close would…	develop COVID-19	5.167	P<0.001	P<0.001	P=0.003
	die from COVID-19	3.422	P<0.001	P<0.001	P<0.001
Chance that…	oneself or another member of one's household losing a job	0.793	0.354	0.588	P<0.001
	one's total household income will decline	-1.912	0.137	0.274	P<0.001
Anticipation of experiencing significantly more intense negative emotion when matters are made worse during a crisis by…	other people	0.517	P<0.001	P<0.001	P<0.001
	the government	0.594	P<0.001	P<0.001	P<0.001
	the media	0.528	P<0.001	P<0.001	P<0.001
	autonomous devices	0.488	P<0.001	P<0.001	P<0.001

Supplementary Table S2: Descriptive Statistics for control variables

	All	Women	Men	Women vs Men
Variables	Mean (sd)			p-values: ttests for difference
Age	40.8	42.9	38.9	<0.001
	(11.4)	(11.9)	(10.6)	
Number of years in college	3.3	3.2	3.3	0.869
	(1.9)	(1.967)	(1.9)	
Political views: liberal	6.1	6.3	5.9	0.019
	(3.025)	(3.1)	(2.9)	
Self-reported cognitive (math) ability	5.8	5.0	6.4	<0.001
	(2.9)	(3.0)	(2.6)	
US Citizen, %	99.3	99.1	99.5	0.391
Race: Caucasian, %	84.4	86.8	82.4	0.019
Hispanic, %	5.8	3.5	7.8	<0.001
Attend Religious services, %	17.8	20.1	15.7	0.027
Relative Household Income:				
Significantly lower, %	10.4	10.6	10.2	0.812
Somewhat lower, %	28.2	29.9	26.7	0.178
About the same, %	43.7	42.9	44.3	0.579
Somewhat higher, %	16.9	15.7	18	0.227
Significantly higher, %	0.9	1.0	0.8	0.594
Smoker, %	17.6	18.3	17.0	0.526
Do not have a job, %	12.5	13.8	11.3	0.157
Work full time, %	75.3	67.7	82	<0.001
Work part time, %	12.2	18.6	6.7	<0.001
**Occupation:**				
Healthcare, %	4.9	7.8	2.4	<0.001
Management, business, and financial operations occupations, %	22.3	18.8	25.3	0.003
Natural resources, construction, and maintenance occupations, %	4.4	2.2	6.3	<0.001
Production, transportation, and material moving occupations, %	3.8	2.9	4.5	0.099
Professional and related occupations -not healthcare, %	27.6	25.5	29.3	0.099
Sales and office occupations, %	21.8	25.5	18.5	0.001
Service occupations -not healthcare, %	4.6	3.9	5.2	0.251
Other, %	10.7	13.3	8.4	0.002
**Education:**				
High school, %	12.5	12.6	12.3	0.877
Some college, %	29.8	33.6	26.4	<0.001
Bachelor's/equivalent, %	45.2	40.7	49.1	<0.001
Masters or above, %	12.5	13.0	12.1	0.581
Mother Education:				

Some high school/less, %	6.6	6.1	7.1	0.455
High School, %	36.9	40.0	34.3	0.022
Some college, %	22.2	23.6	21.0	0.232
Bachelor's/equivalent, %	24.1	20.7	27.1	0.004
Masters or above, %	10.0	9.4	10.6	0.459
Do not know, %	0.1	0.1	0.0	0.284
**Father Education:**				
Some high school/less, %	9.4	10.3	8.6	0.255
High School, %	33.3	35.1	31.7	0.174
Some college, %	19.4	20.0	18.9	0.591
Bachelor's/equivalent, %	23.7	21.0	26.1	0.022
Masters or above, %	13.0	12.6	13.4	0.672
Do not know, %	1.2	1.0	1.4	0.515
ZIP code:				
NY, %	6.9	6.8	6.9	0.930
CA, %	9.1	7.7	10.3	0.077
Sample used in Analysis	1484	690	794	0.007
Wave 1: April 2nd	488	238	250	0.588
Wave 2: April 16th	499	240	259	0.396
Wave 3: April 30th	497	212	285	0.001
Exclusions				
Unmatched ZIP code	4	0	4	
>=2 wrong check questions	3	1	2	
Total number of responses	1491	691	800	

Supplementary Table S3: Fear of COVID-19 (11-point Likert question; Fixed effect linear regression)

	(a)	(b)
Dependent variable:	Afraid of COVID-19	Afraid of COVID-19
Female	.9625***	.6542**
	(.2627)	(.2564)
Wave 2	-.5552***	-.6466***
	(.1313)	(.1433)
Wave 3	-.7139***	-.7745***
	(.1996)	(.2131)
Local Death Rate	-	.0079***
		(.0023)
Cognitive Ability	-	-.0996**
		(.0378)
Liberal	-	.2662***
		(.0361)
Additional controls	No	Yes
State fixed effects	Yes	Yes
Observations	1484	1484
R-squared	.0366	.1316

Standard errors (clustered at the state level) in parentheses. Additional controls included age, age-squared, and indicators for race (Caucasian) and origin (Hispanic), occupation (8 categories), self-reported same or high household income relative to others in one's community, working full time, education level, parents receiving a bachelor's degree, smoking behavior and frequency of attending religious services. *** *p<.01, ** p<.05, * p<.1*

Supplementary Table S4: Separate analyses for preventative measures taken in response to COVID-19: 1) washing hands more frequently, 2) using hand sanitizers or disinfection wipes more frequently, 3) make more effort to avoid touching face (Logit regression)

Dependent variable:	(a) Washed hands	(b) Washed hands	(c) Used hand sanitizers	(d) Used hand sanitizers	(e) Avoid touching face	(f) Avoid touching face
Female	1.3151	1.1294	1.2438	1.1079	1.4831**	1.3105
	(.322)	(.2885)	(.2065)	(.1992)	(.2686)	(.2597)
Afraid of COVID-19	-	1.4115***	-	1.2129***	-	1.2205***
		(.0784)		(.0404)		(.0336)
Wave 2	.9958	1.1830	1.1614	1.3226**	1.2672*	1.4797***
	(.2341)	(.3229)	(.1355)	(.1814)	(.1537)	(.204)
Wave 3	.8408	.9735	1.3923	1.6311**	1.1666	1.3815
	(.2888)	(.3528)	(.3071)	(.3621)	(.2332)	(.2748)
Local Death Rate	1.0104***	1.0069*	1.0028	1.0007	1.0093**	1.0067*
	(.0033)	(.0042)	(.0031)	(.0031)	(.0038)	(.004)
Cognitive Ability	.9027**	.9387	.9429	.9615	1.0427	1.0697**
	(.04)	(.0468)	(.036)	(.0362)	(.0319)	(.0348)
Liberal	1.0700	.9562	1.0709***	1.0156	1.1425***	1.0836***
	(.0552)	(.0592)	(.0279)	(.0282)	(.0319)	(.0299)
Additional controls	Yes	Yes	Yes	Yes	Yes	Yes
State fixed effects	Yes	Yes	Yes	Yes	Yes	Yes
Observations	1242	1242	1461	1461	1442	1442

Odds ratios reported. Standard errors (clustered at the state level) in parentheses. Additional controls included age, age-squared, and indicators for race (Caucasian) and origin (Hispanic), occupation (8 categories), self-reported same or high household income relative to others in one's community, working full time, education level, parents receiving a bachelor's degree, smoking behavior and frequency of attending religious services. ****p<.01, **p<.05, *p<.1*

Supplementary Table S5: Separate analyses for preventative measures taken in response to COVID-19: 1) Cleaning and disinfecting surfaces in home more than usual, 2) Wearing a face mask, 3) Engaging in physical distancing (PD) (Logit regression)

Dependent variable:	(a) Cleaned more	(b) Cleaned more	(c) Worn a facemask	(d) Worn a facemask	(e) Engaged in PD	(f) Engaged in PD
Female	1.5525***	1.4091**	1.0538	.9413	1.6608**	1.4306
	(.2284)	(.215)	(.1281)	(.1285)	(.4028)	(.4138)
Afraid of COVID-19	-	1.2135***	-	1.1888***	-	1.3293***
		(.0227)		(.0245)		(.0894)
Wave 2	.8953	1.0027	7.3309***	9.0008***	.9080	1.1124
	(.1283)	(.1608)	(1.1878)	(1.5596)	(.2626)	(.3291)
Wave 3	1.075	1.2283	12.7076***	16.1669***	.9501	1.1974
	(.1749)	(.2087)	(3.0383)	(3.9878)	(.3139)	(.4177)
Local Death Rate	1.0012	.9992	1.0102*	1.0082	1.0026	.9984
	(.0029)	(.003)	(.0058)	(.0055)	(.0067)	(.0072)
Cognitive Ability	.9807	.9995	.9820	.9982	1.0264	1.0618
	(.0287)	(.0297)	(.0248)	(.0268)	(.0515)	(.0599)
Liberal	1.0733***	1.0167	1.1026***	1.0566**	1.1811***	1.1051**
	(.0235)	(.0225)	(.0265)	(.0256)	(.0386)	(.0441)
Additional controls	Yes	Yes	Yes	Yes	Yes	Yes
State fixed effects	Yes	Yes	Yes	Yes	Yes	Yes
Observations	1478	1478	1476	1476	1170	1170

Odds ratios reported. Standard errors (clustered at the state level) in parentheses. Additional controls included age, age-squared, and indicators for race (Caucasian) and origin (Hispanic), occupation (8 categories), self-reported same or high household income relative to others in one's community, working full time, education level, parents receiving a bachelor's degree, smoking behavior and frequency of attending religious services. ****p<.01, **p<.05, *p<.1*

Supplementary Table S6: Beliefs of financial and health hardships (Fixed effect linear regression)

Dependent variable:	(a)<br>%<br>Job<br>Loss	(b)<br>%<br>Income<br>Loss	(c)<br>%<br>Develop<br>COVID-19	(d)<br>%<br>Die<br>COVID-19
Female	.6182	-2.6648	3.3407***	2.4245**
	(1.2807)	(1.8464)	(1.2238)	(1.0241)
Wave 2	-5.1791***	-7.3799***	-6.1417***	-2.6616***
	(1.4053)	(1.927)	(1.3933)	(.811)
Wave 3	-4.9088***	-6.4673***	-6.1561***	-2.4501***
	(1.8276)	(2.1922)	(1.7764)	(.9032)
Local Death Rate	.0231	.0391	.0329*	.0244
	(.0354)	(.0573)	(.0189)	(.0174)
Cognitive Ability	-.4742	-.5207	-.687***	-.8268***
	(.3271)	(.4628)	(.2482)	(.1394)
Liberal	.3471	.7254	1.4148***	.2491
	(.3695)	(.4429)	(.2697)	(.1903)
Additional controls	Yes	Yes	Yes	Yes
State fixed effects	Yes	Yes	Yes	Yes
Observations	1484	1484	1484	1484
R-squared	.0602	.0833	.0984	.0556

Standard errors (clustered at the state level) in parentheses. Additional controls included age, age-squared, and indicators for race (Caucasian) and origin (Hispanic), occupation (8 categories), self-reported same or high household income relative to others in one's community, working full time, education level, parents receiving a bachelor's degree, smoking behavior and frequency of attending religious services. ****p<.01, **p<.05, *p<.1*

Supplementary Table S7: Expected negative emotional experience (e.g. sadness or anger) in a hypothetical scenario where (a) other people, (b) the government, (c) the media or (d) an autonomous system take actions that make matters worse in a crisis.

	(a)<br>People	(b)<br>Government	(c)<br>Media	(d)<br>Autonomous
Female	.3555***	.4630***	.3846**	.3148**
	(.1263)	(.1426)	(.1546)	(.1256)
Wave 2	-.3385***	-.2408***	-.2832**	-.2000
	(.1195)	(.0897)	(.1101)	(.1524)
Wave 3	-.1623	-.2449	-.2120	-.1320
	(.1627)	(.1732)	(.1789)	(.1931)
Local Death Rate	.0021	.0012	.0018	.0017
	(.002)	(.0024)	(.0021)	(.0019)
Cognitive Ability	-.0109	.0132	-.0078	-.0275
	(.0211)	(.0192)	(.021)	(.0234)
Liberal	.0624***	.1056***	-.0456**	.0291
	(.0208)	(.0252)	(.0203)	(.0181)
Additional controls	Yes	Yes	Yes	Yes
State fixed effects	Yes	Yes	Yes	Yes
Observations	1484	1484	1484	1484
R-squared	.0674	.0999	.0719	.0732

Standard errors (clustered at the state level) in parentheses. Additional controls included age, age-squared, and indicators for race (Caucasian) and origin (Hispanic), occupation (8 categories), self-reported same or high household income relative to others in one's community, working full time, education level, parents receiving a bachelor's degree, smoking behavior and frequency of attending religious services. *** *p<.01, *** *p<.05, ** *p<.1*

1.4 Administered Survey Questions

The following questions will be completed on the computer, and will not be handed out physically.

Section 1:

Section 1.1 (block1)

1. Gender:
    a. Male
    b. Female
2. Age (numeric field)
3. What is your current zip code?
4. Are you
    a. American citizen
    b. Non-American citizen: please specify country
5. Are you Hispanic or Latino?
    a. Yes
    b. No
6. How would you describe yourself?
    a. American Indian or Alaska Native
    b. Asian
    c. Black or African American
    d. Native Hawaiian or Other Pacific Islander
    e. White
    f. Other
7. Do you regularly attend religious services?
    a. Yes
    b. No
8. What is your household income relative to others in your county/city?
    a. Significantly higher
    b. Somewhat higher
    c. About the same
    d. Somewhat lower
    e. Significantly lower
9. What is your height? (numeric field)
10. Are you a smoker?
    a. Yes
    b. No

Section 1.2 (block 2)

11. Do you:
    a. Work at a full-time job
    b. Work at a part-time job?
    c. Do not have a job

12. If you are in (went to) college, how many years of formal education had you completed?
    a. Not applicable
    b. 1 year of college (or equivalent)
    c. 2 years of college (or equivalent)
    d. 3 years of college (or equivalent)
    e. 4 years of college (or equivalent)
    f. 5 years of college (or equivalent)
    g. 6 years of college (or equivalent)
    h. More than 6
13. If you are (were) in college, what is (was) your Major/College? (if more than one pick what you consider to be your primary Major or College).
    a. Economics
    b. Architecture and Urban Studies
    c. Agriculture and Life Science
    d. Business other than Economics
    e. Engineering
    f. Liberal Arts and Human Sciences
    g. Natural Resources and Environment
    h. Science other than Economics
    i. Other major/college
    j. Not applicable
14. How many Economics classes have you taken at the university level?
    a. None
    b. One
    c. Two
    d. Three
    e. Four or more
15. Please indicate the highest level of education YOU completed:
    a. Some high school
    b. High school diploma or equivalent
    c. Some college or associate degree
    d. B.A.
    e. M.A./M.S./M.B.A.
    f. M.D./J.D./PhD
    g. Other
16. What is YOUR current occupation? If you are retired, please list your most recent occupation. (text field)
17. Please indicate the highest level of education your MOTHER completed:
    a. Some high school
    b. High school diploma or equivalent
    c. Some college or associate degree
    d. B.A.
    e. M.A./M.S./M.B.A.
    f. M.D./J.D./PhD
    g. Other

18. What is your MOTHER's current occupation? If she is retired or deceased, please list her most recent occupation. (text field)
19. Please indicate the highest level of education your FATHER completed:
    a. Some high school
    b. High school diploma or equivalent
    c. Some college or associate degree
    d. B.A.
    e. M.A./M.S./M.B.A.
    f. M.D./J.D./PhD
    g. Other
20. What is your FATHER's current occupation? If he is retired or deceased, please list his most recent occupation. (text field)

Section 2:

Section 2.1 (block3)

21. Please describe your political orientation in general, using a scale from 0 to 10, where 0 means you are "complete conservative" and 10 means you are "complete liberal."
22. How willing or unwilling you are to take risks, using a scale from 0 to 10, where 0 means you are "completely unwilling" and 10 means you are "very willing."
23. How willing or unwilling are you to give up something that is beneficial for you today in order to benefit more from that in the future, using a scale from 0 to 10, where 0 means you are "completely unwilling" and 10 means you are "very willing."
24. How willing or unwilling are you to punish someone who treats YOU unfairly, even if there may be costs for you, using a scale from 0 to 10, where 0 means you are "completely unwilling" and 10 means you are "very willing."
25. How willing or unwilling are you to punish someone who treats OTHERS unfairly, even if there may be costs for you, using a scale from 0 to 10, where 0 means you are "completely unwilling" and 10 means you are "very willing."
26. How willing or unwilling are you to give to good causes without expecting anything in return, using a scale from 0 to 10, where 0 means you are "completely unwilling" and 10 means you are "very willing."

How well do the following statements describe you as a person, using a scale from 0 to 10, where 0 means "does not describe me at all," and 10 means "describes me perfectly."

27. When someone does me a favor, I am willing to return it.
28. If I am treated very unjustly, I will take revenge at the first occasion, even if there is a cost to do so.
29. I assume that people have only the best intentions.
30. I am good at math.
31. I tend to postpone tasks even if I know it would be better to do them right away.

Section 2.2 (block 4)

32. For the gambles on each line of the following table, think about the chance of getting each prize being determined by the flip of a coin, for example, for Gamble 3 you would get 32 if the coin comes up "heads" and 8 if it comes up "tails."

    Please choose your favorite gamble below.

Gamble Choice	Probability	Payoff	Probability	Payoff
1	50%	16	50%	16
2	50%	24	50%	12
3	50%	32	50%	8
4	50%	40	50%	4
5	50%	48	50%	0

33. Imagine the following situation: Today you unexpectedly received 1,600 U.S. dollars. How much of this amount would you donate to a good cause? (Values between 0 and 1,600 are allowed)
34. Please think about what you would do in the following situation. You are in an area you are not familiar with, and you realize that you lost your way. You ask a stranger for directions. The stranger offers to take you to your destination.

    Helping you costs the stranger about 40 U.S. dollars in total. However, the stranger says he or she does not want any money from you. You have six presents with you. The cheapest present costs 10 U.S. dollars, the most expensive one costs 60 U.S. dollars. Do you give one of the presents to the stranger as a "thank you" gift?

    Which present do you give to the stranger?

    a. No, would not give a present
    b. The present worth 10 U.S. dollars
    c. The present worth 20 U.S. dollars
    d. The present worth 30 U.S. dollars
    e. The present worth 40 U.S. dollars
    f. The present worth 50 U.S. dollars
    g. The present worth 60 U.S. dollars

Section 3:

Section 3.1 (block 5)

How much do you agree with each of the following statements, using a scale from 0 to 10, where 0 means "do not agree at all," and 10 means "totally agree"?

35. People can generally be trusted
36. Nowadays one can't rely on anyone
37. If one is dealing with strangers, it is better to be careful before trusting them
38. Would you say that people usually…
    a. Try to be helpful
    b. only pursue their own interests
39. Do you believe that most people…
    a. Would exploit you if they had the opportunity
    b. Would try to be fair to you

Section 3.2 (block 6)

How much do you agree with each of the following statements?

Use a scale from 0 to 10, where 0 means "do not agree at all," and 10 means "totally agree"

40. Government can generally be trusted
41. Media can generally be trusted
42. Autonomous systems, for example, artificial intelligence devices can generally be trusted
43. Suppose there is a crisis and other people make a decision or provide information that makes matters worse. To what extent would you experience negative emotions (e.g. sadness or anger) as a result? Use a scale from 0 to 10, where 0 means "Not at all," and 10 means "A great deal."
44. Suppose there is a crisis and the Government makes a decision or provides information that makes matters worse. To what extent would you experience negative emotions (e.g. sadness or anger) as a result? Use a scale from 0 to 10, where 0 means "Not at all," and 10 means "A great deal."
45. Suppose there is a crisis and the Media makes a decision or provides information that makes matters worse. To what extent would you experience negative emotions (e.g. sadness or anger) as a result? Use a scale from 0 to 10, where 0 means "Not at all," and 10 means "A great deal."
46. Suppose there is a crisis and an autonomous system, for example, an artificial intelligence device, makes a decision or provides information that makes matters worse. To what extent would you experience negative emotions (e.g. sadness or anger) as a result? Use a scale from 0 to 10, where 0 means "Not at all," and 10 means "A great deal."

Section 4:

Section 4.1 (block 7)

For the following questions, physical distancing refers to limiting physical contact with people outside household as much as possible.

How much do you agree with each of the following statements, using a scale from 0 to 10, where 0 means "do not agree at all," and 10 means "totally agree"

47. Right now, people in my area engage in physical distancing
48. Right now, people in my area expect me to engage in physical distancing
49. Right now, people in my area expect others to engage in physical distancing
50. Right now, people in my area should engage in physical distancing
51. Physical distancing will slow the spread of a highly infectious disease.
52. I am willing to make personal sacrifices to prevent the spread of coronavirus disease (COVID-19)
53. The COVID-19 outbreak is causing financial stress to me and my family.
54. I have taken the following steps in response to the coronavirus disease (COVID-19). Check all that apply
    - Washed my hands more frequently than usual
    - Used hand sanitizer or disinfecting wipes more frequently than usual
    - Made more of an effort to avoid touching my eyes, nose, mouth
    - Cleaned and disinfected surfaces in my home more than usual
    - Worn a face mask
    - Started working from home
    - Engaged in physical distancing

Section 4.2 (block 8)

55. In the past week, I have purchased more household items and food than usual.
    a. Yes
    b. No

Now, we will ask you some questions about future, uncertain outcomes. In each case, try to think about the whole range of possible outcomes and think about how likely they are to occur during the next 12 months. In some of the questions, I will ask you about the PERCENT CHANCE of something happening. The percent chance must be a number between zero and one hundred. Numbers like 2 or 5 percent may be "almost no chance," 20 percent or so may mean "not much chance," a 45 or 55 percent chance may be a "pretty even chance," 80 percent or so may mean a "very good chance," and a 95 or 98 percent chance may be "almost certain." The percent chance can also be thought of as the NUMBER OF CHANCES OUT OF 100.

56. What do you think is the percent chance that you or another member of your household will lose a job or business due to COVID-19? (numeric field from 0 to 100)
57. What do you think is the percent chance that your total household income will decrease over the next 12 months? (numeric field from 0 to 100)
58. What do think is the percent chance that you, or someone you are close to, will develop COVID-19? (numeric field from 0 to 100)
59. What do think is the percent chance that you, or someone you are close to, will die from COVID-19? (numeric field from 0 to 100)
60. Are you afraid of the COVID-19 pandemic? Please indicate your answer using a scale from 0 to 10, where 0 means “not at all afraid,” and 10 means “very afraid.”

The following check questions added in between some of the survey questions:

1. There are 12 days in a week (True/False)
2. There are two L's in the word "Log" (True/False)
3. Dogs have wings (True/False)
4. Would you rather have $50 or $75?
5. Fish live in water (True/False)

1.5 Summary table for all survey responses

Supplementary Table 8: Summary statistics for all survey responses

Measure	Question no.	Mean	Median	Standard Deviation	Minimum	Maximum
Section 1						
Female, %	1	46.50				
Age	2	40.76	38.00	11.37	20	77
Zip code	3					
New York, %		6.87				
California, %		9.10				
American citizen, %	4	99.33				
Hispanic, %	5	5.80				
Race	6					
American Indian or Alaska Native, %		0.74				
Asian, %		7.28				
Black or African American, %		5.32				
White, %		84.43				
Other, %		2.22				
religious, %	7	17.79				
Income	8					

Significantly lower, %		10.38				
Somewhat lower, %		28.17				
About the same, %		43.67				
Somewhat higher, %		16.91				
Significantly higher, %		0.88				
height (inches)	9	67.88	68.00	3.91	48	79
smoker, %	10	17.59				
Work status	11					
Do not have a job, %		12.47				
Work at a full-time job, %		75.34				
Work at a part-time job, %		12.20				
Years in college	12	3.26	4.00	1.91	0	7
Primary major studied	13					
Agriculture and Life Science, %		0.81				
Architecture and Urban Studies, %		0.54				
Business other than Economics, %		19.00				
Economics , %		2.90				
Engineering, %		7.88				

Liberal Arts and Human Sciences, %		26.62				
Natural Resources and Environment, %		1.42				
Science other than Economics, %		14.22				
Not applicable, %		14.96				
Other, %		11.66				
Number of economics classes taken	14					
None, %		42.25				
One course, %		26.35				
Two courses, %		20.49				
Three courses, %		3.84				
Four course or more, %		7.08				
Education	15					
High school , %		12.47				
Some college or associate degree, %		29.78				
Bachelor degree, %		45.22				
Masters/above, %		12.53				
Occupation (coded from free response)	16					
Healthcare, %		4.92				

Management, business, and financial operations occupations, %		22.30				
Natural resources, construction, and maintenance occupations, %		4.38				
Production, transportation, and material moving occupations, %		3.77				
Professional and related occupations -not healthcare, %		27.56				
Sales and office occupations, %		21.77				
Service occupations -not healthcare, %		4.58				
Other, %		10.71				
Mother education	17					
Some high school or less , %		6.60				
High school , %		36.93				
Some college or associate degree, %		22.24				
Bachelor degree, %		24.12				
Masters/above, %		10.04				
Other, %		0.07				
Mother occupation (free response)	18					
Father education	19					
Some high school or less , %		9.37				
High school , %		33.29				

Some college or associate degree, %		19.41				
Bachelor degree, %		23.72				
Masters/above, %		13.01				
Other, %		1.21				
Father occupation (free response)	20					
Section 2						
Political orientation (10 is complete liberal)	21	6.07	7.00	3.02	0	10
Willingness to take risks (10 is very willing)	22	4.07	4.00	2.45	0	10
Willingness to delay rewards (10 is very willing)	23	7.08	7.00	1.98	0	10
Willingness to punish someone who treats you unfairly (10 is very willing)	24	3.98	4.00	2.71	0	10
Willingness to punish someone who treats others unfairly (10 is very willing)	25	4.62	5.00	2.63	0	10
Willingness to give to good causes (10 is very willing)	26	7.33	8.00	2.40	0	10
Willing to return favor (10 is describes me perfectly)	27	8.75	9.00	1.55	0	10
Will take revenge (10 is describes me perfectly)	28	2.66	2.00	2.68	0	10
Assume that people have only the best intentions (10 is describe me perfectly)	29	5.16	6.00	2.77	0	10
Good at math (10 is describes me perfectly)	30	5.77	6.00	2.85	0	10
Tend to postpone tasks (10 is describes me perfectly)	31	3.80	3.00	2.80	0	10
Unincentivized lottery task	32	2.53	2.00	1.35	1	5

Unincentivized dictator game	33	108.33	50.00	184.34	0	2500
Gift in exchange for help	34	3.27	4.00	1.94	1	6
Section 3						
People can generally be trusted, (10 is totally agree)	35	5.61	6.00	2.61	0	10
One can't rely on anyone, (10 is totally agree)	36	3.94	3.00	2.95	0	10
Its better to be careful before trusting strangers, (10 is totally agree)	37	6.50	7.00	2.59	0	10
People usually try to be helpful (base: only persue their own interests), %	38	64.02				
People would try to be fair (base: exploit you if they had the opportunity), %	39	64.76				
Government can generally be trusted, (10 is totally agree)	40	3.84	4.00	2.57	0	10
Media can generally be trusted, (10 is totally agree)	41	4.03	4.00	2.80	0	10
Autonomous systems can generally be trusted, (10 is totally agree)	42	5.39	5.00	2.42	0	10
Expected negative emotions when other people make matters worse in a crisis (10 is a great deal)	43	6.88	7.00	2.08	0	10
Expected negative emotions when government make matters worse in a crisis (10 is a great deal)	44	7.51	8.00	2.12	0	10
Expected negative emotions when media make matters worse in a crisis (10 is a great deal)	45	7.30	8.00	2.20	0	10
Expected negative emotions when autonomous systems make matters worse in a crisis (10 is a great deal)	46	6.32	6.00	2.30	0	10
Section 4						

People in my area engage in physical distancing (10 is totally agree)	47	8.01	8.00	1.92	0	10
People in my area engage expect me to engage in physical distancing (10 is totally agree)	48	8.34	9.00	1.98	0	10
People in my area engage expect others to engage in physical distancing (10 is totally agree)	49	8.37	9.00	1.83	0	10
People in my area engage should engage in physical distancing (10 is totally agree)	50	9.09	10.00	1.73	0	10
Physical distancing will slow the spread of a highly infectious disease (10 is totally agree)	51	8.77	10.00	1.89	0	10
Willing to make personal sacrifices to prevent the spread of COVID-19 (10 is totally agree)	52	8.75	10.00	2.02	0	10
COVID-19 outbreak is causing financial stress to me and my family (10 is totally agree)	53	5.29	6.00	3.39	0	10
Measures taken in response to COVID-19	54					
Washed my hands more frequently than usual, %		94.20				
Used hand sanitizer or disinfecting wipes more frequently than usual, %		78.23				
Made more of an effort to avoid touching my eyes, nose, mouth, %		82.75				
Cleaned and disinfected surfaces in my home more than usual, %		72.51				
Worn a face mask , %		51.75				
Started working from home, %		54.65				
Engaged in physical distancing, %		93.80				

Purchased more household items and food than usual in the past week, %	55	37.47				
Probabilistic beliefs about the likelihood of job loss	56	26.58	20.00	28.13	0	100
Probabilistic beliefs about the likelihood of income loss	57	41.49	37.50	33.57	0	100
Probabilistic beliefs about the likelihood of oneself or someone close developing COVID-19	58	32.43	25.00	25.78	0	100
Probabilistic beliefs about the likelihood of oneself or someone close dying from COVID-19	59	10.56	5.00	15.95	0	100
Afraid of COVID-19 (10 is very afraid)	60	5.93	7.00	3.02	0	10
